# The Caregiver Transportation Scale Measures the Impact of Driving Cessation on Caregivers

**DOI:** 10.1093/geroni/igaa057.086

**Published:** 2020-12-16

**Authors:** Michel Bedard, Shauna Fossum, Dwight Mazmanian, Hello Møller, Jessica Lowey

**Affiliations:** Lakehead University, Thunder Bay, Ontario, Canada

## Abstract

Driving cessation can impact retiring drivers, but we often neglect to consider its effect on caregivers. Caregivers may have to deal with important changes when someone they care for ceases driving, but we have few means to quantify these changes. Hence, we aimed to develop the Caregiver Transportation Scale (CTS) to measure this impact. We developed a bank of positive and negative questions, then pre-tested it with a small sample of caregivers (N = 11), leading to reduction and refinement of the questions. We pilot-tested this set of questions with a larger caregiver sample (phase 2; N = 73). Preliminary validation of the tool relied on correlation analyses with the Zarit Burden Interview (ZBI) and relevant demographic questions. For phase 2, the mean caregiver age was 61.9 (SD = 10.23, range 20-83); most caregivers were female (80.8%) and were adult-child caregivers (61.7%). The final version of the CTS contains 24 items. Internal consistency (Cronbach’s alpha) was .90. The mean caregiver score was 64.75 (SD = 16.76, range 24-98); about 1/3 of caregivers’ scores fell above the middle possible score of 72. The scores were positively correlated with the Zarit Burden Interview (r = .74, p < .001) and negatively correlated with the availability of others to help with driving responsibilities (r = -.36, p = .002). The CTS has the potential to help inform, develop, and evaluate services for caregivers who provide transportation support for older adults who ceased driving. However, further validation is required before we recommend its use.

